# Angiosarcoma of the Right Atrium with Extension to SVC and IVC Presenting with Complete Heart Block and Significant Pericardial Effusion

**DOI:** 10.1155/2016/3173069

**Published:** 2016-03-27

**Authors:** Hossein Vakili, Isa Khaheshi, Mehdi Memaryan, Shooka Esmaeeli

**Affiliations:** ^1^Cardiovascular Research Center, Modarres Hospital, Shahid Beheshti University of Medical Sciences, Tehran, Iran; ^2^Student Scientific Research Center (SSRC), Tehran University of Medical Sciences (TUMS), Tehran, Iran

## Abstract

Primary cardiac neoplasms are particularly unusual. Angiosarcoma is the most frequently seen histological subtype and is described by its infiltrating and damaging nature. Inappropriately, primary cardiac angiosarcoma is often missed as a preliminary diagnosis because of its scarcity. We present a 29-year-old previously healthy man with complete heart block and pericardial effusion who was finally diagnosed with angiosarcoma of the right atrium with extension to SVC and IVC.

## 1. Case Presentation

A 29-year-old man, with no remarkable past medical history, presented to our emergency unit with chief complaint of weakness and class III functional dyspnea 2 weeks earlier.

He had no chest pain, cold sweat, or episode of syncope. He was alert and hemodynamically stable, with blood pressure of 110/80, heart rate of 48, respiratory rate of 20, and temperature of 37.2°C and oxygen saturation of 92% in the ambient air.

On physical examination, heart sounds obviously diminished; however, he had no significant pulsus paradoxus. The remainder of his examination was not notable.

His electrocardiogram (ECG) showed complete heart block with a ventricular rate of 52 and no ST segment or T changes.

**Figure 1:**
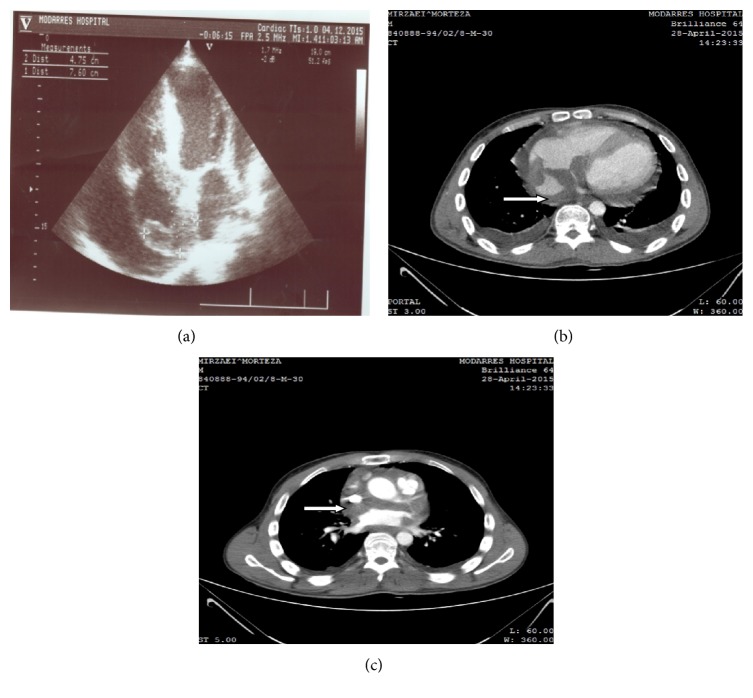
(a) TTE of patient which showed a large echogenic mass (76 mm*∗*47 mm) in the right atrium which extended toward atrioventricular junction and septal leaflet of tricuspid valve and pericardial effusion around LV, RV, and RA with no tamponade physiology. (b and c) Spiral CT-scan of the chest with and without IV contrast which showed an infiltrative circumferential mass lesion (*white arrow*) involving the right walls of the right atrium and also interatrial septum with extension to the upper part of interventricular septum.

A comprehensive transthoracic echocardiography (TTE) showed ejection fraction (EF): 55%, mild tricuspid regurgitation, trivial mitral regurgitation, systolic pulmonary artery pressure (SPAP) of 40 mmHg, a large echogenic mass (76 mm*∗*47 mm) in the right atrium which extended toward atrioventricular junction and septal leaflet of tricuspid valve, and moderate pericardial effusion (13 mm) around LV, RV, and RA with no tamponade physiology (Figure 1(a)).

Following TTE findings, transesophageal echocardiography was done which revealed a large homogenous lobulated mass in the right atrium with attachment to interatrial septum and extension to superior vena cava, inferior vena cava, and AV junction (maximum size: 77 mm*∗*45 mm); he underwent spiral CT-scan of the chest with and without IV contrast which showed an infiltrative mass lesion involving the right lateral and posterior walls of the right atrium and also interatrial septum with extension to the upper part of interventricular septum. In addition, the most upper part of IVC in suprahepatic and the most distal part of SVC were involved but not obstructed (Figures 1(b) and 1(c)).

Due to exacerbation of his dyspnea and increasing of pericardial effusion, he underwent therapeutic pericardiocentesis. The pericardial fluid was relatively clear and serous.

Cytology of the pericardial fluid was negative for malignancy; also, smear and culture of the pericardial fluid were negative for any infection including tuberculosis.

According to consultation with cardiac surgery department, a mass excision surgery was planned for him.

He underwent open heart surgery but due to extension and invasion of the tumor to the RA, SVC, and IVC, excision was not possible and only surgical tissue biopsy was taken. The result of the tissue biopsy was angiosarcoma. Spiral CT-scan of the chest, abdomen, and pelvis revealed no metastasis.

We referred him to oncology service for starting chemotherapy after all.

## 2. Discussion

Primary cardiac neoplasms are particularly unusual. Angiosarcoma is the most frequently seen histological subtype and is described by its infiltrating and damaging nature. Inappropriately, primary cardiac angiosarcoma is often missed as a preliminary diagnosis because of its scarcity [1–3].

The most common complaint of the patients is dyspnea; this indiscriminate symptomatology contributes to the trouble in diagnosing such circumstances. More precise clinical findings are typically apparent later in the progression of the disease and dependent on the degree of infiltration within the heart walls [3, 4].

Early diagnosis of primary cardiac angiosarcoma remains challenging. Echocardiography is the cornerstone of assessing cardiac tumors. Transesophageal echocardiography has 97% sensitivity in identifying cardiac masses [5].

The prognosis for primary cardiac angiosarcoma is not good, with mean survival of 3.8 ± 2.5 months without surgical resection; however, it was suggested that tumor size and the extent of regional expansion of the tumor were correlated with time of survival [4, 6, 7].

Therapy is not uniform and the precise benefit of adjunctive chemotherapy and/or radiation is not still definite. The challenges of therapy include the infrequency of this disease, the usually progressive stage at diagnosis, and its aggressive course [7, 8].

We presented a young male with pericardial effusion and complete heart block finally diagnosed as primary cardiac angiosarcoma. To our knowledge, this is the first case of primary angiosarcoma with this relatively rapid course and clinical presentation that involved both SVC and IVC.

This case underscores the importance of the echocardiography as a suitable imaging modality for detecting cardiac tumors including cardiac angiosarcoma and emphasizes the importance of primary cardiac tumor in differential diagnosis of manifestations including complete heart block and pericardial effusion. This is particularly essential for general cardiologists and emergency unit clinicians to be familiar with these uncommon circumstances.
